# High expression of GPR176 predicts poor prognosis of gastric cancer patients and promotes the proliferation, migration, and invasion of gastric cancer cells

**DOI:** 10.1038/s41598-023-36586-3

**Published:** 2023-06-08

**Authors:** Yu Zhang, Xinliang Gu, Feilong Zhu, Yang Li, Yuejiao Huang, Shaoqing Ju

**Affiliations:** 1 Medical School of Nantong University, Nantong University, Nantong, China; 2 Department of Laboratory Medicine, Affiliated Hospital of Nantong University, Xisi Road, NO.20, Nantong, China; 3 Research Center of Clinical Medicine, Affiliated Hospital of Nantong University, Nantong, China; 4 Department of Medical Oncology, Affiliated Hospital of Nantong University, Xisi Road, NO.20, Nantong, China

**Keywords:** Cancer, Computational biology and bioinformatics, Molecular biology

## Abstract

G-protein-coupled receptors (GPCRs) are the most prominent family of cell surface receptors, which can regulate various biological functions and play an essential role in many diseases. GPR176 is a member of the GPCRs family and has been rarely studied in cancer. We aim to investigate the diagnostic and prognostic value of GPR176 in gastric cancer (GC) and explore its potential mechanism. Through the TCGA database and real-time quantitative PCR, we found that the expression level of GPR176 was significantly increased in GC and had good value in the diagnosis and prognosis of GC. Vitro experiments revealed that GPR176 could promote the proliferation, migration, and invasion of GC cells and may be involved in regulating multiple tumors and immune-related signaling pathways. In addition, we found that GPR176 is associated with GC immune infiltration and may affect the immune efficacy of GC patients. In summary, the high GPR176 expression level was associated with poor prognosis, more robust immune infiltration, and worse immunotherapy efficacy in GC patients, suggesting that GPR176 may be an immune-related biomarker for GC that can promote the proliferation, migration, and invasion of GC cells.

## Introduction

Gastric cancer (GC) is one of the most severe cancers in the world. According to statistics, there are more than one million new cases of GC worldwide in 2020, with the fourth and fifth highest incidence and mortality rates worldwide, respectively^1^.

The risk factors for GC include H. pylori infection, age, and high salt intake, with H. pylori infection being the leading cause of GC^2^. The decline in GC incidence and mortality over the last half-century has been due to continued prevention efforts, decreased prevalence of H. pylori, and improved food storage practices^3^. However, in recent years, the incidence of GC has been increasing in both low and high-risk countries^4,5^, a phenomenon that may be related to the increasing use of antibiotics and acid suppressants^6,7^. Most GC patients are already at an advanced stage when detected, and most have metastatic disease, thus missing the opportunity for radical resection, which results in a low five-year survival rate^2,8^. Therefore, searching for new biomarkers with good sensitivity and specificity to identify patients with GC and provide personalized treatment is urgent.

G-protein-coupled receptors (GPCRs) are the most prominent family of cell surface receptors involved in regulating various biological functions, including intercellular communication, neuronal transmission^9^. Meanwhile, because of their central role in various diseases and physiological processes, GPCRs are also the therapeutic targets of nearly one-third of clinically marketed drugs^10–12^. Many GPCRs remain unknown physiologically, so exploring their function and role remains a hot topic^9^. GPR176, a member of the GPCRs family, was initially identified in the human brain and has been little researched in cancer, only being found to be associated with heart-free acid transcription in breast cancer cell lines^13,14^. The role of GPR176 in GC, however, remains unknown.

In this study, GPR176 was significantly increased in GC by The Cancer Genome Atlas (TCGA) database and validated by relevant experiments. Subsequently, we evaluated the diagnostic and prognostic value of GPR176 in GC and determined its correlation with clinicopathological parameters. Additionally, we discovered that GPR176 regulates a variety of tumor and immune-related signaling pathways and can promote the proliferation, migration, and invasion of GC cells. Furthermore, we analyzed the correlation between GPR176 and tumor immune infiltration and the possible response of patients with different GPR176 expression levels to immunotherapy. In this study, we provide more theoretical support for the potential use of GPR176 to diagnose and prognosis GC, offering new possibilities for clinical differentiation of GC patients.

## Materials and methods

### Data collection

The Cancer Genome Atlas (TCGA; http://cancergenome.nih.gov/) database contains 373 GC tissue samples and 32 adjacent normal tissue samples, and clinical information on these GC tissue samples is available from the UC Santa Cruz Xena Browser (http://xena.ucsc.edu/). The Gene expression matrix for GC cell lines was obtained from the CCLE dataset (https://sites.broadinstitute.org/ccle). All data were analyzed by R software (https://www.r-project.org/).

### Human tissue specimens

All tissue specimens, including 50 GC tissues and adjacent non-tumor tissues were collected from the Department of Pathology, Affiliated Hospital of Nantong University. All specimens were diagnosed as GC by pathologists and immediately frozen in liquid nitrogen after resection and transferred to a −80 °C refrigerator for long-term storage. In this study, all patients with GC were clinically diagnosed and did not receive radiotherapy or chemotherapy before. This study got approval from the ethics committee of the local hospital (ethics review report number: 2018-L055).

### Cell culture and cell transfection

The Chinese Academy of Sciences (Shanghai, China) provided human gastric epithelial cells (GES-1) as well as two GC cell lines (HGC-27, MKN-1). Both HGC-27 and MKN-1 are epithelial-like adherent cells. HGC-27 was derived from undifferentiated GC tissue, whereas MKN-1 was derived from gastric lymph nodes, both identified using STR. We cultured these cells in RPMI-1640 medium (Corning, USA) with 10% fetal bovine serum (Gibco, USA) at 37 °C with 5% CO_2_ in an incubator. Lipo3000 (ThermoFisher Scientific, USA) was used to transfer plasmids into cells.

### Cell counting Kit‑8 (CCK-8) and clone formation assays

3 × 10^3^ cells were inoculated in each well of a 96-well plate and incubated for 2 h at 37 °C after 12 h with 10 μL of CCK-8 assay (Biosharp, China) and the optical density (OD) values at 450 nm and 630 nm were read by an enzyme-labeled instrument^15^. The test was then carried out every 24 h for three consecutive times. The OD values at 450 nm minus 630 nm were calculated. 1000 cells were inoculated in each well of a six-well plate and changed every four days^16^. 2 weeks later 4% paraformaldehyde was fixed, and photographed after crystalline violet staining.

### Transwell assay for cell migration and invasion

5 × 10^4^ cells were inoculated in the upper chamber and a complete medium containing 20% fetal bovine serum was added to the lower chamber^17^. The number of cells entering the lower chamber was counted to reflect the migration ability of the tumor cells. Before the invasion assay, a layer of stromal gel was placed in the upper chamber and 8 × 10^4^ cells were inoculated into the lower chamber after the stromal gel had solidified^18^, the number of cells entering the lower chamber could reflect the invasion ability of the cells.

### Total RNA extraction, reverse transcription, and Real-time quantitative PCR (RT-qPCR)

TRIzol Reagent (Invitrogen, Germany) was used to extract the total RNA of tissue specimens, and Revert Aid RT Reverse Transcription Kit (ThermoFisher Scientific, USA) was used to produce cDNA. RT-qPCR was performed on ABI QuantStudio5 with 10 μL ChamQ Universal SYBR qPCR Master Mix (Vazyme Biotech Co., Ltd, China), 0.5 μL primers (10 μM), 4 μL enzyme-free Water and 5 μL cDNA. GAPDH was used as an internal reference, and the expression of GPR176 was calculated by the 2^−∆∆CT^ method. The sequences of primer used in this study were GPR176-F: GATGGTCTTCATCTTGTGTAGC, GPR176-R: CTCCCTGTACTGACCACATTAC; GAPDH-F: AGAAGGCTGGGGCTCATTTG, GAPDH-R: GCAGGAGGCATTGCTGATGAT.

### The Human Protein Atlas

Comparison of GPR176 protein expression between Stomach adenocarcinoma (STAD) and normal gastric tissues was performed using the immunohistochemistry images on The Human Protein Atlas (https://www.proteinatlas.org)^19^.

### Construction of the protein–protein interaction (PPI) network

The protein–protein interaction (PPI) network of GPR176 was constructed using GeneMANIA (http://genemania.org/), which can use a very large set of functional association data to find other genes associated with the target gene^20^.

### Functional enrichment analysis

#### Gene Set Enrichment Analysis (GSEA)

We divided GC patients into high and low expression groups based on the expression level of GPR176 and identified the pathways enriched to GPR176 in GC by GSEA v4.2.3 downloaded from GSEA (https://www.gsea-msigdb.org/gsea/index.jsp). The gene sets ‘c2.cp.kegg.v7.5.1.symbols.gmt’ from the Molecular Signature Database (MSigDB) was used as the reference for GSEA, and the P-value < 0.05 and q-value < 0.25 were considered with significant differences.

#### Gene Ontology (GO) and Kyoto Encyclopedia of Genes and Genomes (KEGG) enrichment analyses

The R package “tidyverse”, “clusterProfiler”, and “org.Hs.eg.db” were used to perform GO and KEGG analyses on GPR176, and the P-value < 0.05 and q-value < 0.05 were considered with significant differences.

### Tumor immune infiltration analysis

Tumor immune estimation resource (TIMER) is a comprehensive resource for systematical analysis of immune infiltrates across diverse cancer types and TIMER2.0 (http://timer.cistrome.org/) is an updated version of TIMER^21^. We analyzed the correlation between GPR176 and six different immune cells using TIMER2.0.

The CIBERSORT method was used to estimate the abundance of tumor-infiltrating immune cells from the gene expression profiles in all STAD samples^22^.

The single-sample gene set enrichment analysis (ssGSEA) method from the R package “GSVA” was used to calculate the degree of infiltration of 28 immune cell types between two groups based on the published expression levels of 28 immune cell genomes^23,24^.

ESTIMATE is a method that uses gene expression to infer the ratio of stromal to immune cells in tumor specimens^25^. We used this method to assess the immune score, stromal score, ESTIMATE score, and tumor purity of each STAD sample.

We downloaded the normalized pan-cancer dataset from the UCSC database and extracted GPR176, 150 genes from the five classes of immune pathways (chemokine, receptor, MHC, Immunoinhibitor, Immunostimulator), and 60 genes from the two classes of immune checkpoint pathways (Inhibitory, Stimulatory)^26^ expression data for marker genes in individual samples. Then, we performed the correlation analysis by Spearman's analysis.

### Prediction of immunotherapy efficacy

Tumor Immune Dysfunction and Exclusion (TIDE, http://tide.dfci.harvard.edu/) allows for the calculation of a TIDE score that negatively correlates with the efficacy of immunotherapy by modeling the mechanism of tumor immune escape through the expression profile of genes in the tumor. We calculated the TIDE score for STAD patients using TIDE and compared it to the expression level of GPR176.

The Cancer Immunome Database (TCIA, https://tcia.at/home) provides results of comprehensive immunogenomic analyses for 20 solid cancers from TCGA and other data sources^27^. We predicted the immunogenicity of patients with different expression levels of GPR176 to immunotherapy using TCIA.

### Association Analysis of GPR176 with Tumor Mutational Burden (TMB), Microsatellite Instability (MSI), Mismatch Repair (MMR), and DNA Methyltransferases

The TMB data, MSI data, and the expression of MMR genes and four methyltransferases were obtained from the TCGA database. The correlations between the expression level of GPR176 and them were analyzed using Spearman’s analysis.

### Data analysis

All data in the study were first checked for normality, and were expressed as the mean ± standard deviation. A t-test or Mann–Whitney U test was used to analyze the expression level of GPR176 in two dependent groups. Paired t-tests or Wilcoxon test was used for paired samples and the expression level of GPR176 in GC cells were compared using one-way analysis of variance (ANOVA). The R package “survival” was used to perform survival analysis in two groups, and the log-rank test was used to assess the significance of prognostic differences between different groups. Univariate and multivariate Cox regression analyses were performed to assess the relationship of GPR176 and other clinicopathological parameters with the prognosis of GC patients. The R package “regression modeling strategies (rms)” was used to plot nomograms. All analyses of correlation were performed using Spearman’s analysis. The P-value < 0.05 and r > 0.3 was considered significant and positively correlated.

## Results

### The expression level of GPR176 in GC and its diagnostic and prognostic value

**Figure 1:**
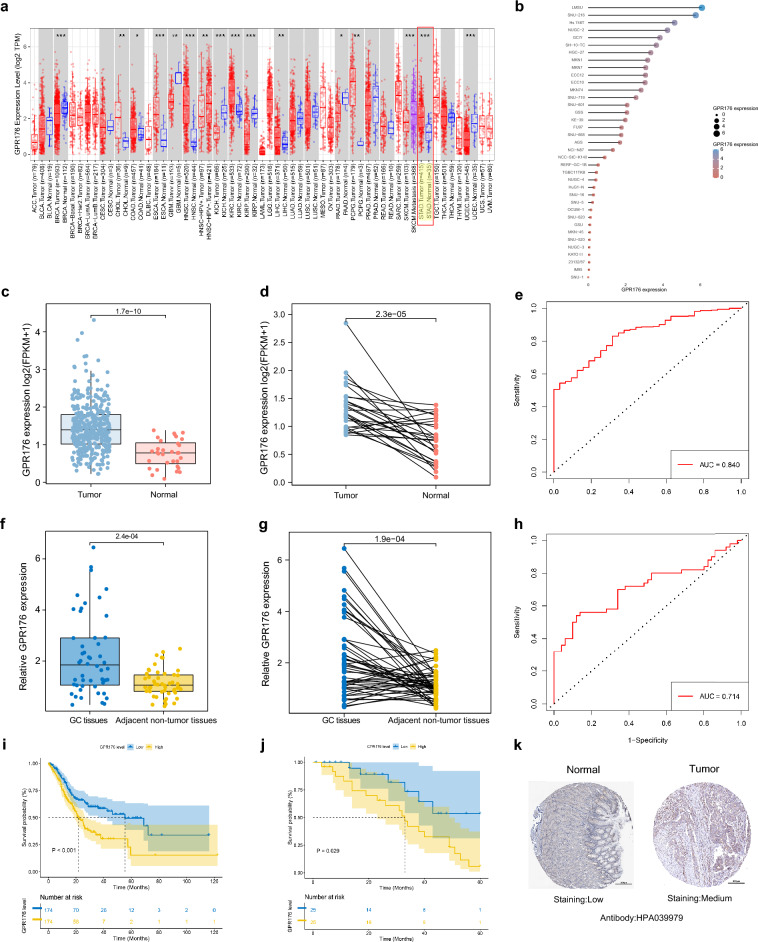
The expression level of GPR176 in GC and its diagnostic and prognostic value. (**a**) The expression levels of GPR176 in pan-cancer; (**b**) The expression levels of GPR176 in GC cells from the CCLE dataset; (**c**) The expression level of GPR176 in TCGA STAD database (Tumor = 373, Normal = 32); (**d**) The expression level of GPR176 in paired GC samples in TCGA STAD database (N = 27); (**e**) ROC curve of GPR176 in TCGA STAD database; (**f**) The expression level of GPR176 in GC tissues (GC tissues = 50, Adjacent non-tumor tissues = 50); (**g**) The expression level of GPR176 in paired GC tissues (N = 50); (**h**) ROC curve of GPR176 in GC tissues; (**i**) Kaplan–Meier survival curve of GPR176 in TCGA STAD database; (**j**) Kaplan–Meier survival curve of GPR176 in GC tissues; (**k**) Comparison of immunohistochemistry images of GPR176 between GC and normal gastric tissues based on the Human Protein Atlas (*P < 0.05, **P < 0.01, ***P < 0.001).

As shown in Fig. 1a, GPR176 expression levels were significantly different in Breast invasive carcinoma (BRCA), Esophageal carcinoma (ESCA), Head and Neck squamous cell carcinoma (HNSC), Kidney Chromophobe (KICH), Kidney renal clear cell carcinoma (KIRC), Kidney renal papillary cell carcinoma (KIRP), STAD, and Uterine Corpus Endometrial Carcinoma (UCEC), suggesting an essential role in cancer. For follow-up research, we focused on GC and found high GPR176 expression in various GC cell lines (Fig. 1b). To further explore the diagnostic value of GPR176 in GC, we analyzed the expression levels of GPR176 in the TCGA STAD database and found that it was significantly higher in GC tissues (n = 373) than in adjacent normal tissues of cancer (n = 32; P < 0.001; Fig. 1c, d). Receiver operating characteristic curve (ROC) analysis showed that the area under the curve (AUC) of GPR176 was 0.840, demonstrating its good diagnostic efficacy in GC (Fig. 1e). Then, we collected 50 pairs of GC tissues and their adjacent non-tumor tissues. The RT-qPCR results showed that the expression level of GPR176 was significantly increased in GC tissues (P < 0.001; Fig. 1f, g) and the AUC was 0.714 (Fig. 1h), which was consistent with the results of the TCGA STAD database. Kaplan–Meier survival analysis showed that the overall survival (OS) was lower in the high GPR176 group compared to the low GPR176 group (Fig. 1i, j). In addition, immunohistochemistry showed deeper staining levels of GPR176 in GC tissues than in normal gastric tissues, indicating higher protein levels of GPR176 in GC tissues (Fig. 1k). Taken together, the increased expression of GPR176 in GC correlated with poor prognosis, suggesting that it may be a critical gene that could be used to differentiate GC patients clinically.

### Correlation of GPR176 with clinicopathological parameters and is an independent prognostic factor for GC

By analyzing the correlation between GPR176 and clinicopathological parameters in GC, we found that GPR176 was significantly associated with the depth of infiltration and survival status. At the same time, there was no significant relationship with other pathological parameters (see Supplementary Table S1 online). Then, we performed univariate and multivariate Cox regression analysis to determine the clinical prognostic value of GPR176 in GC. Univariate analyses identified GPR176 (hazard ratio (HR) = 1.83, P < 0.001), residual tumor (HR = 3.40, P < 0.001), Stage (HR = 1.84, P = 0.001), M stage (HR = 2.33, P = 0.004), N stage (HR = 1.57, P = 0.007), T stage (HR = 1.65, P = 0.018) and age (HR = 1.60, P = 0.007) were all significantly associated with OS in GC patients (see Supplementary Fig. S1a online, Table 1). Multivariate analysis showed that GPR176 (HR = 1.80, P = 0.003), residual tumor (HR = 2.81, P < 0.001), and age (HR = 1.55, P = 0.027) were independent prognostic factors for GC (see Supplementary Fig. S1b online, Table 1).

**Table 1:** Univariate and multivariate Cox regression analysis of GPR176 in GC.

Characteristics	Univariate analysis			Multivariate analysis		
	Hazard.Ratio	95%CI	P-value	Hazard.Ratio	95%CI	P-value
Age	1.6	1.14–2.24	0.007**	1.55	1.05–2.27	0.027*
Gender	1.35	0.95–1.94	0.095			
M	2.33	1.31–4.13	0.004**	1.52	0.74–3.12	0.259
MSI_status	0.98	0.7–1.39	0.928			
N	1.57	1.13–2.19	0.007**	1.49	0.92–2.43	0.107
Residual_tumor	3.4	2.13–5.41	< 0.001***	2.81	1.61–4.93	< 0.001***
Stage	1.84	1.3–2.62	0.001**	1.16	0.64–2.11	0.629
T	1.65	1.09–2.49	0.018*	1.08	0.63–1.84	0.788
GPR176	1.83	1.31–2.56	< 0.001***	1.8	1.23–2.64	0.003**

*P < 0.05, **P < 0.01, ***P < 0.001.

### Establishing nomogram for predicting prognosis of OS for GC patients

**Figure 2:**
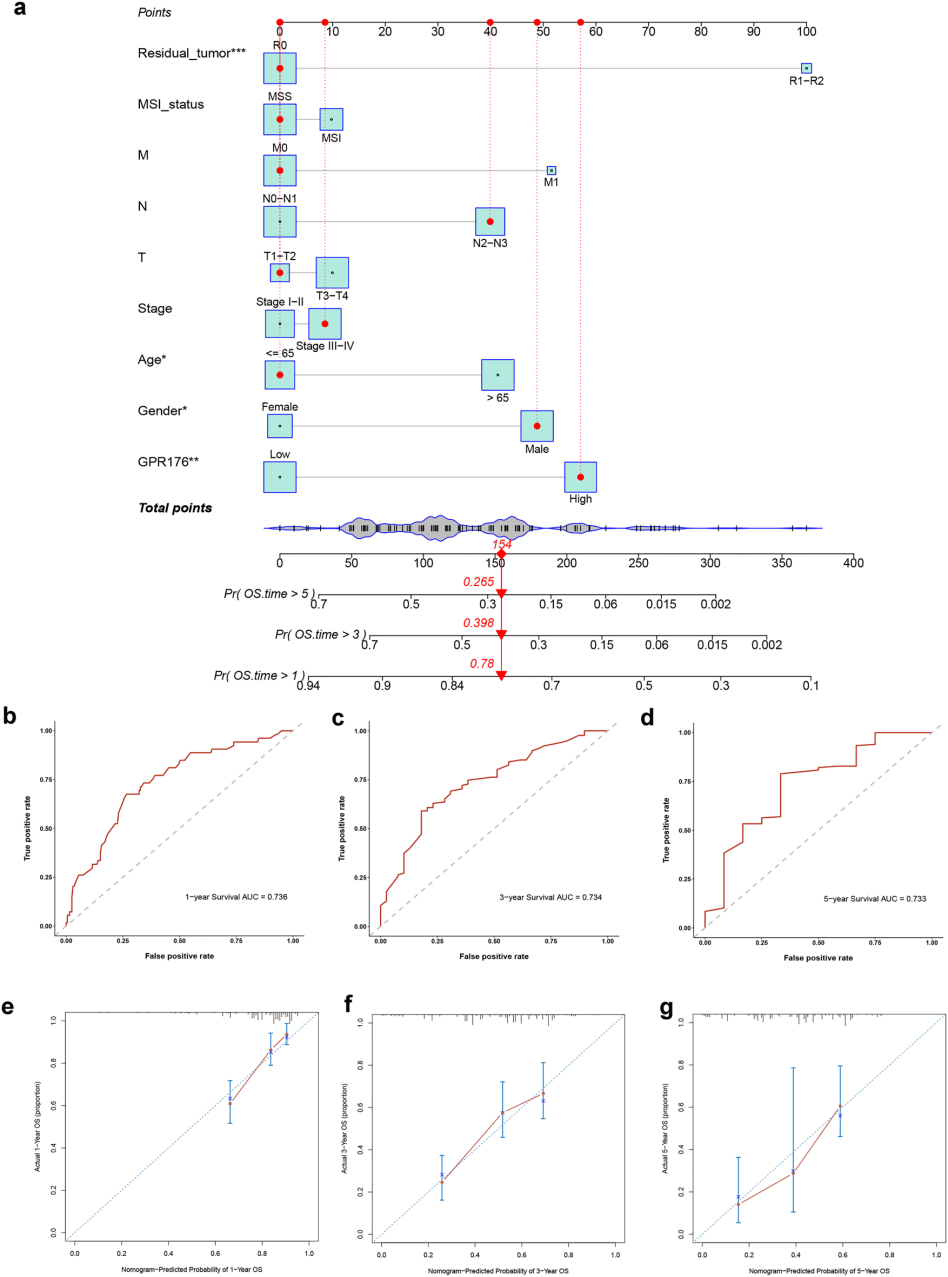
Establishing nomogram for predicting prognosis of OS for GC patients. (**a**) Nomogram for predicting one-, three-, and five-year OS for GC patients in TCGA; (**b-d**) One-, three-, and five-year ROC curves of the established nomogram; (**e–g**) One-, three-, and five-year calibration plots of the established nomogram (*P < 0.05, **P < 0.01, ***P < 0.001).

**Figure 3:**
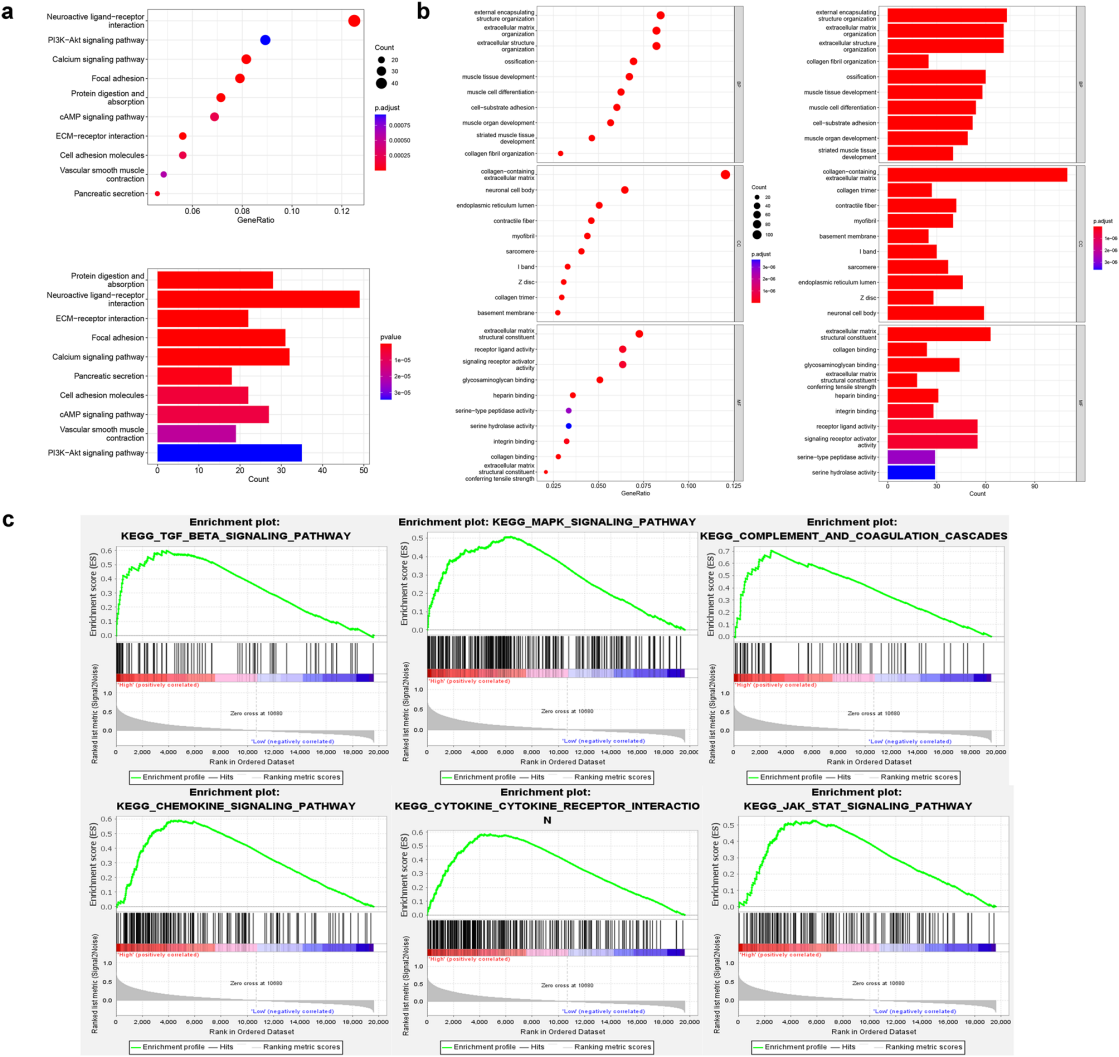
Functional and pathway prediction of GPR176 in GC. (**a**) Top ten enriched pathways based on KEGG analysis; (**b**) Top ten enriched pathways of BP, CC, and MF based on GO analysis; (**c**) Enriched pathways in the high GPR176 group based on GSEA.

To predict the prognostic OS of GC patients, we constructed a Nomogram prognostic model with clinicopathological parameters and GPR176. In this model, a score is defined for each risk factor, and we can predict the OS of the patient based on the total score (the sum of the scores of all risk factors) for each patient: the higher the total score, the lower the OS (Fig. 2a). In addition, the AUC of the 1-year, 3-year, and 5-year ROC curves of the line graph prognostic model were 0.736, 0.734, and 0.733, respectively (Fig. 2b-d), and the calibration curves showed a high degree of fit between the actual and predicted OS without deviation from the reference line (Fig. 3e–g). The above results indicate that the Nomogram prognostic model we constructed has good accuracy and confidence in predicting the OS of GC patients.

### Functional and pathway prediction of GPR176 in GC

To investigate the potential mechanisms by which GPR176 affects GC, we performed KEGG biological pathway enrichment analysis and GO gene function enrichment analysis on the differentially expressed genes according to GPR176 expression levels. KEGG analysis showed that the genes were significantly enriched in the pathways of Protein digestion and absorption, Neuroactive ligand-receptor Interaction, and Extracellular matrix-receptor interaction (Fig. 3a). The results of GO analysis showed that Biological Process (BP) was enriched considerably in internal encapsulating structure organization, extracellular matrix organization, and extracellular structure organization. In Cellular Component (CC), collagen-containing extracellular matrix, collagen trimer, and contractile fiber were significantly enriched, while in Molecular Function (MF), extracellular matrix structural constituent, collagen binding, and glycosaminoglycan binding were significantly enriched (Fig. 3b). GSEA showed that compared to the GPR176 low expression group, the GPR176 high expression group was enriched considerably in two cancer-related signaling pathways: TGF_BETA_SIGNALING_PATHWAY, MAPK_SIGNALING_PATHWAY, and four immune-related signaling pathways: COMPLEMENT_AND_COAGULATION_CASCADES, CHEMOKINE_SIGNALING_PATHWAY, CYTOKINE_CYTOKINE_RECEPTOR_INTERACTION, and JAK_STAT_SIGNALING_PATHWAY (Fig. 3c, see Supplementary Table S2 online). It suggested that GPR176 plays a role in GC progression and tumor immunity.

### GPR176 promotes GC cell proliferation, migration, and invasion abilities

**Figure 4:**
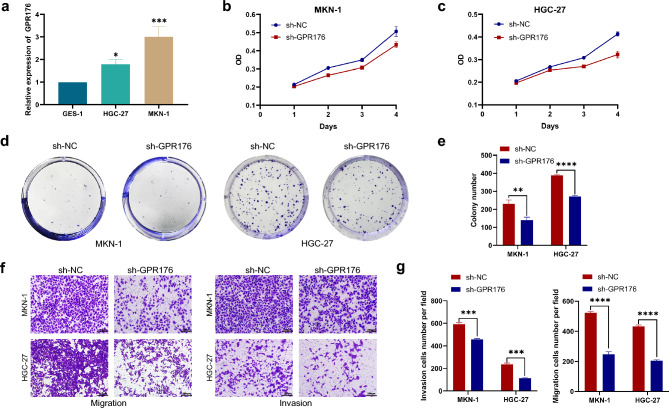
GPR176 promotes GC cell proliferation, migration, and invasion abilities. (**a**) The expression levels of GPR176 in GC cells; (**b,c**) CCK8 assay in MKN-1 and HGC-27 after knockdown of GPR176; (**d,e**) Colony assay in MKN-1 and HGC-27 after knockdown of GPR176; (**f,g**) Transwell assay in MKN-1 and HGC-27 after knockdown of GPR176 (*P < 0.05, **P < 0.01, ***P < 0.001, ****P < 0.0001).

To investigate whether GPR176 is involved in regulating GC progression, we verified its impact on GC cell proliferation, migration, and invasion abilities in vitro. We first detected the expression level of GPR176 in GC cells, and the results showed that it was significantly increased in HGC-27 and MKN-1 (Fig. 4a). CCK8 and clone formation assays showed that the knockdown of GPR176 significantly decreased the proliferation ability of GC cells (Fig. 4b-e). When GPR176 was knocked down, GC cells showed a considerable reduction in migration and invasion abilities (Fig. 4f, g). The above results suggest that GPR176 can promote the proliferation, migration, and invasion of GC cells.

### Correlation of GPR176 with TMB and MSI and construction of PPI networks

To explore the intrinsic mechanisms of GPR176 involvement in the regulation of GC, we constructed a PPI network of GPR176 using the GeneMANIA database (see Supplementary Fig. S2a online). It has been reported that TMB and MSI are closely related to tumor progression and have the potential to be tumor biomarkers^28,29^. We found a significant negative correlation between GPR176 and TMB and MSI in GC, suggesting that GPR176 may influence GC progression by regulating TMB and MSI (see Supplementary Fig. S2b online, c).

### Correlation of GPR176 with the MMR genes and DNA methylation

To further understand the role and mechanism of GPR176, we assessed the relationship between the expression levels of GPR176 and five MMR genes through the TCGA database. The results showed that GPR176 was significantly associated with MLH1, MSH6, and PMS2 in GC (see Supplementary Fig. S3a online). In tumor cells, aberrant DNA methylation is closely associated with tumor progression^30^. The expression levels of GPR176 in GC were positively correlated with two methyltransferases (see Supplementary Fig. S3b online), suggesting that GPR176 might influence GC progression by regulating DNA methylation and MMR.

### Correlation of GPR176 with immune microenvironment and immune infiltration in GC

**Figure 5:**
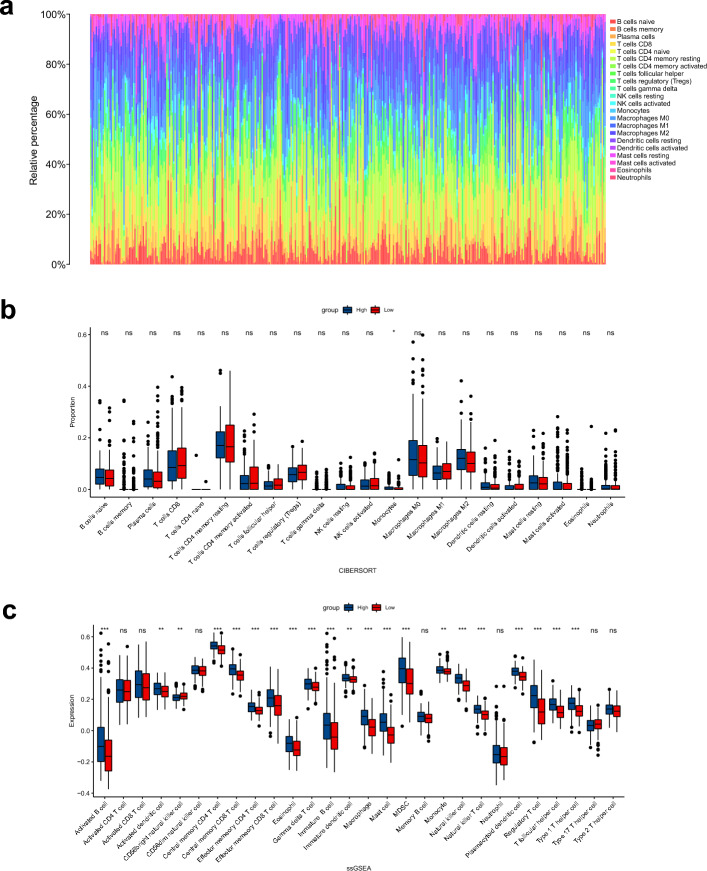
Correlation of GPR176 with immune microenvironment and immune infiltration in GC. (**a**) The proportion of 22 kinds of immune cells in STAD tumor samples; (**b**) Proportion of immune cells between the high GPR176 group and low GPR176 group; (**c**) Expression of immune cells between the high GPR176 group and low GPR176 group (*P < 0.05, **P < 0.01, ***P < 0.001).

**Figure 6:**
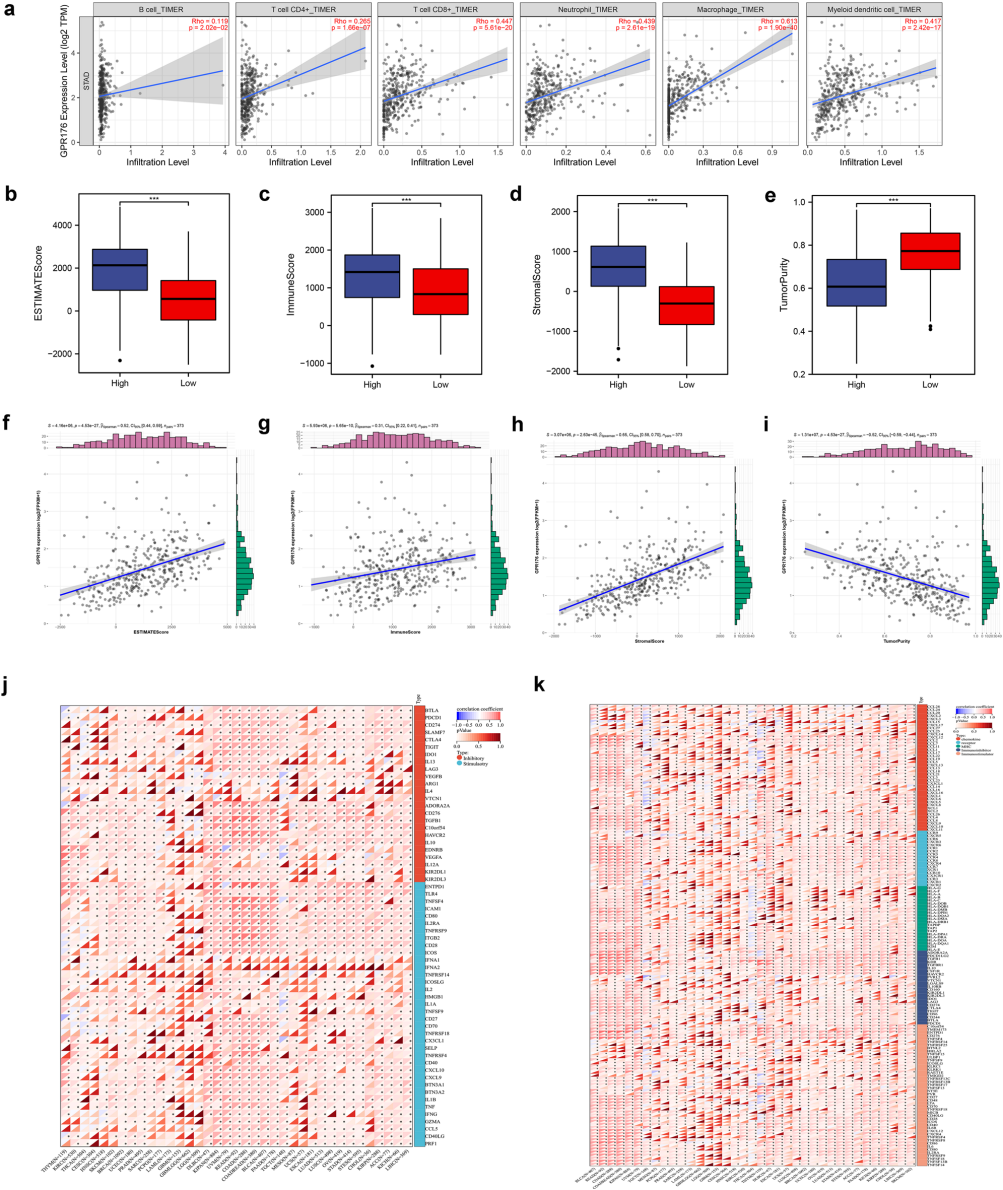
Correlation of GPR176 with immune microenvironment and immune infiltration in GC. (**a**) GPR176 was positively correlated with tumor-infiltrating immune cells in STAD by TIMER; (**b-e**) Comparison of ESTIMATE score, Immune score, Stromal score, and tumor purity between the high GPR176 group and low GPR176 group; (**f-i**) Correlation of the expression of GPR176 with ESTIMATE score, Immune score, Stromal score, and tumor purity; (**j**) Correlation analysis of GPR176 expression with the expression of immune checkpoint genes in pan-cancer; (**k**) Correlation analysis of GPR176 expression with the expression of immunomodulatory genes in pan-cancer (*P < 0.05, **P < 0.01, ***P < 0.001).

To investigate the correlation between the expression level of GPR176 and the immune microenvironment, we analyzed the proportion of tumor immune cell subpopulations using the CIBERSORT algorithm and constructed a profile of 22 immune cell types in GC samples (Fig. 5a). CIBERSORT analysis showed a higher proportion of Monocytes in the high GPR176 expression group (Fig. 5b). In contrast, ssGSEA analysis showed that Activated B cells, Activated dendritic cells, Central memory CD4 T cells, Central memory, CD8 T cell, Effector memeory CD4 T cell, Effector memeory CD8 T cell, Eosinophil, Gamma delta T cell, Immature B cell, Immature dendritic cell Macrophage, Mast cell, MDSC, Monocyte, Natural killer cell, Natural killer T cell, Plasmacytoid dendritic cell, Regulatory T cell, T follicular helper cell, and Type 1 T helper cell were significantly higher in the high GPR176 expression group (Fig. 5c), indicating that the high GPR176 expression group has stronger immune infiltration than the low GPR176 expression group. Then, we assessed the potential relationship between the expression levels of GPR176 and GC tumor-infiltrating immune cells using the TIMER database. The results showed that the expression level of GPR176 was associated with CD8 + T cells (r = 0.447, P = 5.61e−20), Neutrophils (r = 0.439, P = 2.61e−19), Macrophages (r = 0.613, P = 1.90e−40), and dendritic cells (r = 0.417, P = 2.42e−17) were significantly positively correlated (Fig. 6a). We later analyzed the immune scores of GC samples by the ESTIMATE algorithm. We found that the high GPR176 expression group had higher stromal, immune, and ESTIMATE scores and lower tumor purity than the low GPR176 expression group (Fig. 6b-e). In addition, the Spearman correlation analysis showed that GPR176 expression levels were significantly positively correlated with stromal, immune, and ESTIMATE scores and negatively correlated with tumor purity (Fig. 6f-i). We further analyzed the correlation of GPR176 with common immune checkpoint genes and immune regulatory genes in tumors. According to our results, GPR176 correlated significantly with immune checkpoint genes and immunomodulatory genes in GC (Fig. 6j, k), indicating that GPR176 modulates immune checkpoint genes and immunomodulatory genes to regulate immunity.

### Immunotherapeutic response prediction

**Figure 7:**
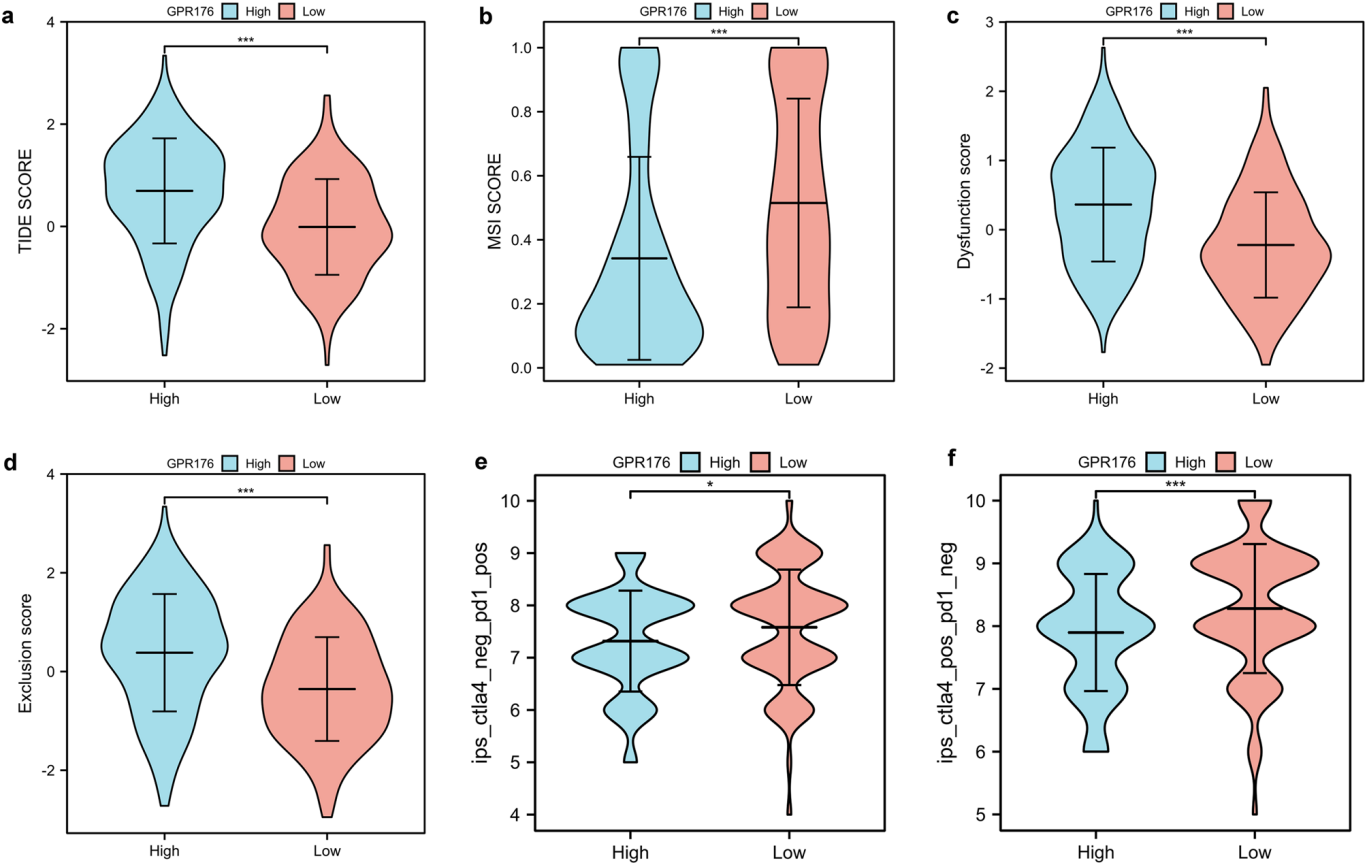
Immunotherapeutic response prediction (**a-d**) Comparison of TIDE score, MSI score, Dysfunction score, and Exclusion score between the high GPR176 group and low GPR176 group based on TIDE database; (**e, f**) Comparison of PD1 score, CTLA4 score between the high GPR176 group and low GPR176 group based on TCIA database (*P < 0.05, ***P < 0.001).

Next, using the TIDE database, we assessed the possible response to immunotherapy in the high and low GPR176 expression groups. The results showed that the high GPR176 expression group had a higher TIDE score, T-cell dysfunction score, T-cell exclusion score, and lower MSI score (Fig. 7a-d), suggesting that the high GPR176 expression group may have a higher rate of tumor immune escape and less effective after receiving immunotherapy. Additionally, we assessed the immunological properties of GPR176 in GC using the TCIA database. The results showed that the low GPR176 group was significantly more immunogenic to immunotherapy with CTLA4 and PD1 than the high GPR176 group (Fig. 7e, f). In conclusion, GC patients with high GPR176 expression are less effective for immunotherapy, while CTLA4 and PD1 may be potential immunotherapeutic targets for patients with low GPR176 expression.

## Discussion

Recently, it has been found that the expression level of GPR176 was increased in colorectal cancer (CRC) and may promote CRC progression via inhibition of mitochondrial autophagy^31^. In addition, GPR176 was identified as a cancer-associated fibroblast marker specific to ovarian cancer and could help guide the prognostic assessment of ovarian cancer^32^. Moreover, the protein expression of GPR176 was significantly increased in breast cancer and it may serve as a potential biomarker to indicate poor prognosis of breast cancer as well as a potential target for gene therapy^33^. These findings reveal a potentially significant role for GPR176 in cancers, suggesting that it may be able to promote the malignant progression of a wide range of cancers, and perhaps has an equally indispensable role in GC. In this study, the bioinformatics analysis of GC tissues showed that GPR176 expression was significantly higher than expected. Its high expression was associated with a lower overall survival rate and had a good diagnostic and prognostic value. A previous study identified GPR176 as a potential biomarker for predicting prognosis and immune infiltration in STAD, but it was not validated using experiments^34^. Using RT-qPCR, we validated this finding and found that it was the same. Correlation analysis of clinicopathological parameters showed that high expression of GPR176 was significantly associated with the depth of infiltration and the survival status, and univariate and multifactorial analyses suggested that GPR176 might be an independent prognostic factor for GC. Additionally, we constructed a Nomogram prognostic model of GPR176 and clinicopathological parameters to predict the prognostic OS of GC patients. The above results suggest that GPR176 may be a new potential biomarker for the diagnosis and prognosis of GC.

However, the specific mechanisms of GPR176 in GC have not been investigated. We found that GPR176 can promote the proliferation, migration, and invasion abilities of GC cells in vitro, indicating that high expression of GPR176 in GC may promote the malignant progression of GC. Furthermore, we predicted and constructed the PPI network of GPR176, identified many genes interacting with GPR176, and analyzed the functional pathways associated with GPR176 and the signaling pathways enriched to GPR176 by KEGG, GO, and GSEA analysis. These findings provide insight into the role of GPR176 in GC and provide ideas for future research.

Notably, the tumor microenvironment, which includes stromal cells, fibroblasts, endothelial cells, and immune cells, is complex and evolving^35^, and the ongoing interactions between the tumor microenvironment and tumor cells are closely associated with tumor progression^36,37^. Futhermore, the acquisition and maintenance of tumor proliferation, apoptosis, and invasion are closely related to Tumor microenvironment (TME)^36^. In addition, immune cells have an essential role in TME. In CRC, researchers have found that patients with stage I or stage II cancers that lack T-cell infiltration undergo recurrence within five years, while stage III patients who do have T-cell infiltration have a longer disease-free survival time^38^. Thus, finding biomarkers relating to TME and immune cells is crucial to developing new cancer therapies. Besides, we found a stronger immune infiltration in the high GPR176 expression group by CIBERSORT and ssGSEA analysis and a significant positive correlation between GPR176 expression levels and CD8^+^ T cells, Neutrophils, Macrophages, and dendritic cells using the TIMER database, which suggests that it may influence GC progression by regulating immune cells. Immunotherapy is a massive breakthrough in cancer treatment as it aims to eliminate tumor cells by boosting their defenses^39^. However, immune checkpoints can maintain immune tolerance, and tumor cells can use immune checkpoints to escape immune surveillance to promote the malignant progression of tumors^40,41^. Tumor immunotherapy has become increasingly important with the emergence of immune checkpoint inhibitors in recent years^29^. In this study, we found that GPR176 was significantly associated with various immune checkpoint genes in GC. Furthermore, TIDE and TCIA databases were used to obtain immunological properties of GPR176 in GC. The low GPR176 expression group showed greater immunogenicity to CTLA4 and PD1 immunotherapy and possibly better efficacy.

In conclusion, although our study demonstrates the great potential of GPR176 for the diagnosis of GC, there are still some limitations to the current study. Firstly, most of our studies are based on the transcriptional level, and the detection of its protein level is also essential if it is to be better applied to the clinical diagnosis of GC. Secondly, liquid biopsy, as an emerging technique in tumor diagnosis, can greatly reduce the difficulty of diagnosis^42^. If the expression level of GPR176 mRNA in blood can be detected using RT-qPCR and its expression level is different between GC patients and healthy controls, it will certainly provide a great convenience and help in the diagnosis of GC in the future.

## Conclusions

In summary, we first found that high expression of GPR176 in GC can predict poor prognosis in GC patients and is closely related to immune infiltration and efficacy. In addition, GPR176 can promote the proliferation, migration, and invasion of GC cells in vitro. Furthermore, it is significantly related to tumor-related pathways and immune-related pathways, showing its potential as a biomarker for GC and providing new possibilities for clinical screening of GC in the future.

## Supplementary Information

Supplementary Information.

## Data Availability

The data used in the current study are available from the corresponding author on reasonable request.

## Abbreviations

GC: Gastric cancer
GPCRs: G-protein-coupled receptors
TCGA: The Cancer Genome Atlas
GES-1: Gastric epithelial cells
CCK-8: Cell counting Kit‑8
OD: Optical density
RT-qPCR: Real-time quantitative PCR
STAD: Stomach adenocarcinoma
PPI: Protein-protein interaction
GSEA: Gene Set Enrichment Analysis
GO: Gene Ontology
KEGG: Kyoto Encyclopedia of Genes and Genomes
TIMER: Tumor immune estimation resource
ssGSEA: Single-sample gene set enrichment analysis
TIDE: Tumor Immune Dysfunction and Exclusion
TCIA: The Cancer Immunome Database
TMB: Tumor Mutational Burden
MSI: Microsatellite Instability
MMR: Mismatch Repair
ANOVA: One-way analysis of variance
rms: Regression modeling strategies
BRCA: Breast invasive carcinoma
ESCA: Esophageal carcinoma
HNSC: Head and Neck squamous cell carcinoma
KIRC: Kidney renal clear cell carcinoma
KIRP: Kidney renal papillary cell carcinoma
UCEC: Uterine Corpus Endometrial Carcinoma
ROC: Receiver operating characteristic curve
AUC: Area under the curve
OS: Overall survival
BP: Biological Process
CC: Cellular Component
MF: Molecular Function
CRC: Colorectal cancer
HR: Hazard ratio

## References

[1] Sung H (2021). Global Cancer Statistics 2020: GLOBOCAN estimates of incidence and mortality worldwide for 36 cancers in 185 countries. CA Cancer J. Clin..

[2] Smyth EC, Nilsson M, Grabsch HI, van Grieken NC, Lordick F (2020). Gastric cancer. Lancet.

[3] Howson CP, Hiyama T, Wynder EL (1986). The decline in gastric cancer: Epidemiology of an unplanned triumph. Epidemiol. Rev..

[4] Arnold M (2020). Is gastric cancer becoming a rare disease? A global assessment of predicted incidence trends to 2035. Gut.

[5] Heer EV, Harper AS, Sung H, Jemal A, Fidler-Benaoudia MM (2020). Emerging cancer incidence trends in Canada: The growing burden of young adult cancers. Cancer.

[6] Anderson WF (2018). The changing face of noncardia gastric cancer incidence among US non-Hispanic whites. J. Natl. Cancer Inst..

[7] Camargo MC (2011). Divergent trends for gastric cancer incidence by anatomical subsite in US adults. Gut.

[8] Zong L, Abe M, Seto Y, Ji J (2016). The challenge of screening for early gastric cancer in China. Lancet.

[9] Goto K (2017). G-protein-coupled receptor signaling through Gpr176, Gz, and RGS16 tunes time in the center of the circadian clock [Review]. Endocr. J..

[10] Wang T (2020). Identification and functional characterisation of N-linked glycosylation of the orphan G protein-coupled receptor Gpr176. Sci. Rep..

[11] Santos R (2017). A comprehensive map of molecular drug targets. Nat. Rev. Drug Discov..

[12] Hauser AS, Attwood MM, Rask-Andersen M, Schiöth HB, Gloriam DE (2017). Trends in GPCR drug discovery: New agents, targets and indications. Nat. Rev. Drug Discov..

[13] Doi M (2016). Gpr176 is a Gz-linked orphan G-protein-coupled receptor that sets the pace of circadian behaviour. Nat. Commun..

[14] Schultz DJ (2018). Transcriptomic response of breast cancer cells to anacardic acid. Sci. Rep..

[15] Gao N (2020). The role of TRPV1 ion channels in the suppression of gastric cancer development. J Exp. Clin. Cancer Res..

[16] Liu JZ (2019). Rafoxanide promotes apoptosis and autophagy of gastric cancer cells by suppressing PI3K/Akt/mTOR pathway. Exp. Cell Res..

[17] Ma C (2020). Circular RNA hsa_circ_0004872 inhibits gastric cancer progression via the miR-224/Smad4/ADAR1 successive regulatory circuit. Mol. Cancer..

[18] Ma S (2021). CircHAS2 promotes the proliferation, migration, and invasion of gastric cancer cells by regulating PPM1E mediated by hsa-miR-944. Cell Death Dis..

[19] Asplund A, Edqvist PH, Schwenk JM, Pontén F (2012). Antibodies for profiling the human proteome-The Human Protein Atlas as a resource for cancer research. Proteomics.

[20] Montojo J, Zuberi K, Rodriguez H, Bader GD, Morris Q (2014). GeneMANIA: Fast gene network construction and function prediction for Cytoscape. F1000Res.

[21] Li T, *et al.* TIMER2.0 for analysis of tumor-infiltrating immune cells. *Nucleic Acids Res.* **48**(W1), W509–W514 (2020). 10.1093/nar/gkaa407 PMC7319575 32442275

[22] Newman AM (2015). Robust enumeration of cell subsets from tissue expression profiles. Nat. Methods..

[23] Bindea G (2013). Spatiotemporal dynamics of intratumoral immune cells reveal the immune landscape in human cancer. Immunity.

[24] Hänzelmann S, Castelo R, Guinney J (2013). GSVA: gene set variation analysis for microarray and RNA-seq data. BMC Bioinform..

[25] Yoshihara K (2013). Inferring tumour purity and stromal and immune cell admixture from expression data. Nat. Commun..

[26] Thorsson V (2018). The immune landscape of cancer. Immunity.

[27] Charoentong P (2017). Pan-cancer immunogenomic analyses reveal genotype-immunophenotype relationships and predictors of response to checkpoint blockade. Cell Rep..

[28] Kok M, Chalabi M, Haanen J (2019). How I treat MSI cancers with advanced disease. ESMO Open..

[29] Addeo A, Friedlaender A, Banna GL, Weiss GJ (2021). TMB or not TMB as a biomarker: That is the question. Crit. Rev. Oncol. Hematol..

[30] Nishiyama A, Nakanishi M (2021). Navigating the DNA methylation landscape of cancer. Trends Genet..

[31] Tang J (2023). GPR176 promotes cancer progression by interacting with G protein GNAS to restrain cell mitophagy in colorectal cancer. Adv. Sci..

[32] Zeng L, Wang X, Wang F, Zhao X, Ding Y (2022). Identification of a gene signature of cancer-associated fibroblasts to predict prognosis in ovarian cancer. Front. Genet..

[33] Yun, W.J., et al. Oncogenic roles of GPR176 in breast cancer: a potential marker of aggressiveness and a potential target of gene therapy. *Clin. Transl. Oncol.* (2023). 10.1007/s12094-023-03174-w PMC10462518 37079213

[34] Ni L (2023). GPR176 Is a biomarker for predicting prognosis and immune infiltration in stomach adenocarcinoma. Mediators Inflamm..

[35] Hinshaw DC, Shevde LA (2019). The tumor microenvironment innately modulates cancer progression. Cancer Res..

[36] Xiao Y, Yu D (2021). Tumor microenvironment as a therapeutic target in cancer. Pharmacol. Ther..

[37] Merlo LM, Pepper JW, Reid BJ, Maley CC (2006). Cancer as an evolutionary and ecological process. Nat. Rev. Cancer..

[38] Mlecnik B (2011). Histopathologic-based prognostic factors of colorectal cancers are associated with the state of the local immune reaction. J. Clin. Oncol..

[39] Zhang Y, Zhang Z (2020). The history and advances in cancer immunotherapy: Understanding the characteristics of tumor-infiltrating immune cells and their therapeutic implications. Cell Mol. Immunol..

[40] Kalbasi A, Ribas A (2020). Tumour-intrinsic resistance to immune checkpoint blockade. Nat. Rev. Immunol..

[41] Chen L, Flies DB (2013). Molecular mechanisms of T cell co-stimulation and co-inhibition. Nat. Rev. Immunol..

[42] Pantel K, Alix-Panabières C (2017). Liquid biopsy in 2016: Circulating tumour cells and cell-free DNA in gastrointestinal cancer. Nat. Rev. Gastroenterol. Hepatol..

