# Supplementary material for: High expression of GPR176 predicts poor prognosis of gastric cancer patients and promotes the proliferation, migration, and invasion of gastric cancer cells
Source: Sci Rep. 2023 Jun 8;13:9360. doi: 10.1038/s41598-023-36586-3 (PMC10250322; doi:10.1038/s41598-023-36586-3)
Supplement: Supplementary file 1 — Supplementary Information. [file 41598_2023_36586_MOESM1_ESM.docx]

**High expression of GPR176 predicts poor prognosis of gastric cancer patients and promotes the proliferation, migration, and invasion of gastric cancer cells**

**Yu Zhang** **^1, 2, 3†^,** **Xinliang Gu ^1, 2, 3†^, Feilong Zhu ^1^, Yang Li ^1, 2, 3^, Yuejiao Huang ^1, 4*^**, **Shaoqing Ju^2*^**

^1^Medical School of Nantong University, Nantong University, Nantong, China.

^2^Department of Laboratory Medicine, Affiliated Hospital of Nantong University, Nantong, China.

^3^Research Center of Clinical Medicine, Affiliated Hospital of Nantong University, Nantong, China.

^4^Department of Medical Oncology, Affiliated Hospital of Nantong University, Nantong, China.

**^†^**Yu Zhang and Xinliang Gu should be considered joint first authors.

**Supplementary Tables**

**Supplementary Table 1**. Clinicopathological analysis of GPR176.

| Parameter |  | No. of patients | GPR176(high) | GPR176(low) | Pearson χ2 | P-value |
| --- | --- | --- | --- | --- | --- | --- |
| Gender | male | 225 | 114 | 111 | 0.113 | 0.737 |
|  | female | 123 | 60 | 63 |  |  |
| Age（year） | <= 65 | 156 | 81 | 75 | 0.983 | 0.612 |
|  | > 65 | 186 | 91 | 95 |  |  |
|  | NA | 6 | 2 | 4 |  |  |
| T | T1-T2 | 90 | 35 | 55 | 9.452 | 0.009 |
|  | T3-T4 | 254 | 135 | 119 |  |  |
|  | NA | 4 | 4 | 0 |  |  |
| N | N0-N1 | 195 | 92 | 103 | 4.395 | 0.111 |
|  | N2-N3 | 143 | 74 | 69 |  |  |
|  | NA | 10 | 8 | 2 |  |  |
| M | M0 | 311 | 152 | 159 | 1.485 | 0.476 |
|  | M1 | 22 | 13 | 9 |  |  |
|  | NA | 15 | 9 | 6 |  |  |
| Stage | I-II | 156 | 72 | 84 | 2.612 | 0.271 |
|  | III-IV | 180 | 94 | 86 |  |  |
|  | NA | 12 | 8 | 4 |  |  |
| Residual_tumor | R0 | 285 | 140 | 145 | 2.28 | 0.32 |
|  | R1-R2 | 29 | 13 | 16 |  |  |
|  | NA | 34 | 21 | 13 |  |  |
| MSI_status | MSS | 235 | 125 | 110 | 2.949 | 0.086 |
|  | MSI | 113 | 49 | 64 |  |  |
| Living_status | Alive | 203 | 87 | 116 | 9.943 | 0.002 |
|  | Death | 145 | 87 | 58 |  |  |

**Supplementary Table 2.** Enriched pathways in the high GPR176 group based on GSEA.

| **NAME** | **NES** | **NOM p-val** | **FDR q-val** |
| --- | --- | --- | --- |
| KEGG_TGF_BETA_SIGNALING_PATHWAY | 2.156855 | 0 | 0.003465257 |
| KEGG_MAPK_SIGNALING_PATHWAY | 2.1515868 | 0 | 0.003033548 |
| KEGG_COMPLEMENT_AND_COAGULATION_CASCADES | 2.087914 | 0 | 0.003532314 |
| KEGG_CHEMOKINE_SIGNALING_PATHWAY | 2.078218 | 0 | 0.004114469 |
| KEGG_CYTOKINE_CYTOKINE_RECEPTOR_INTERACTION | 1.9613044 | 0.002114165 | 0.006626431 |
| KEGG_JAK_STAT_SIGNALING_PATHWAY | 1.8818617 | 0 | 0.011462519 |

**Supplementary Figures**

**Figure S1**


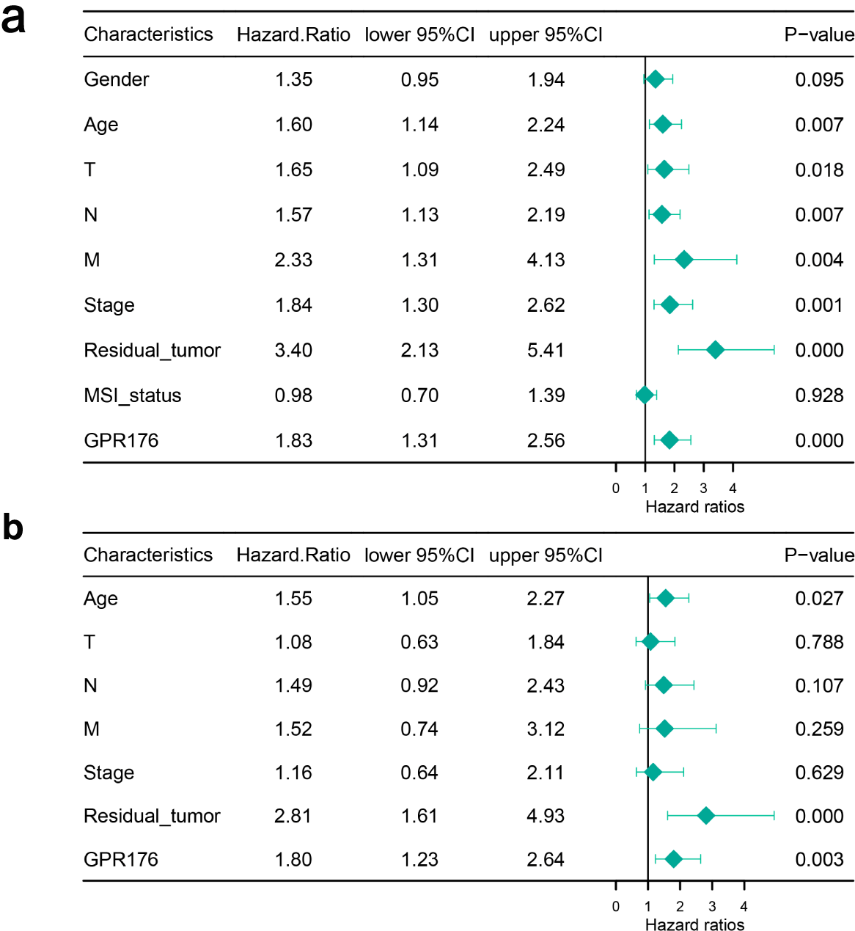


**Supplementary Figure 1.** Univariate and multivariate Cox regression analysis of GPR176 in GC. (a,b) The forest plots showed the predictive power of GPR176 for GC in univariate and multivariate Cox regression analysis.

**Figure S2**

**
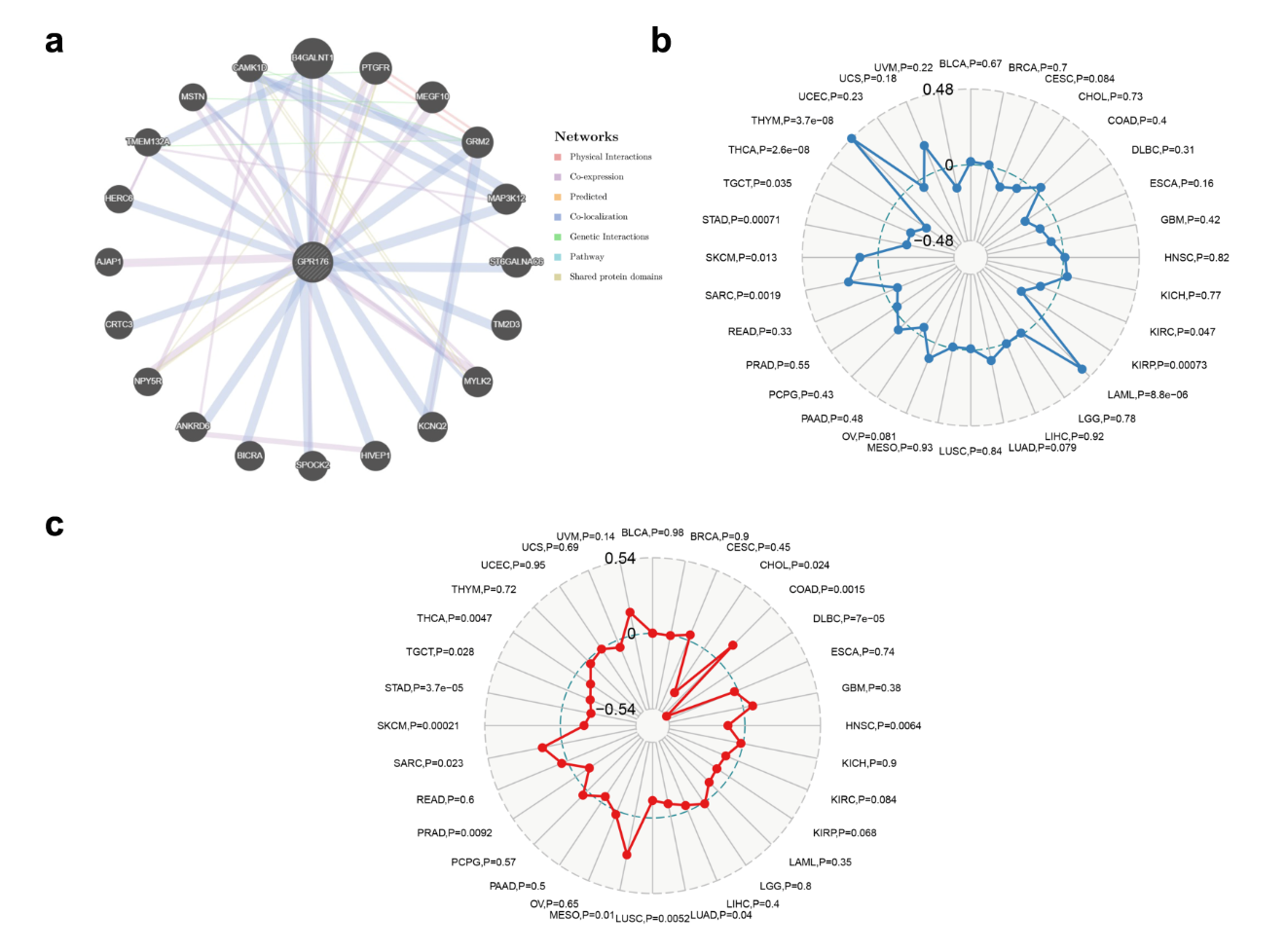
**

**Supplementary Figure 2.** Correlation of GPR176 with TMB and MSI and construction of PPI networks. (a) Protein-protein network view in the GeneMANIA dataset showing the interaction networks of GPR176; (b) The correlation analysis between GPR176 expression and TMB in pan-cancer; (c) The correlation analysis between GPR176 expression and MSI in pan-cancer.

**Figure S3**


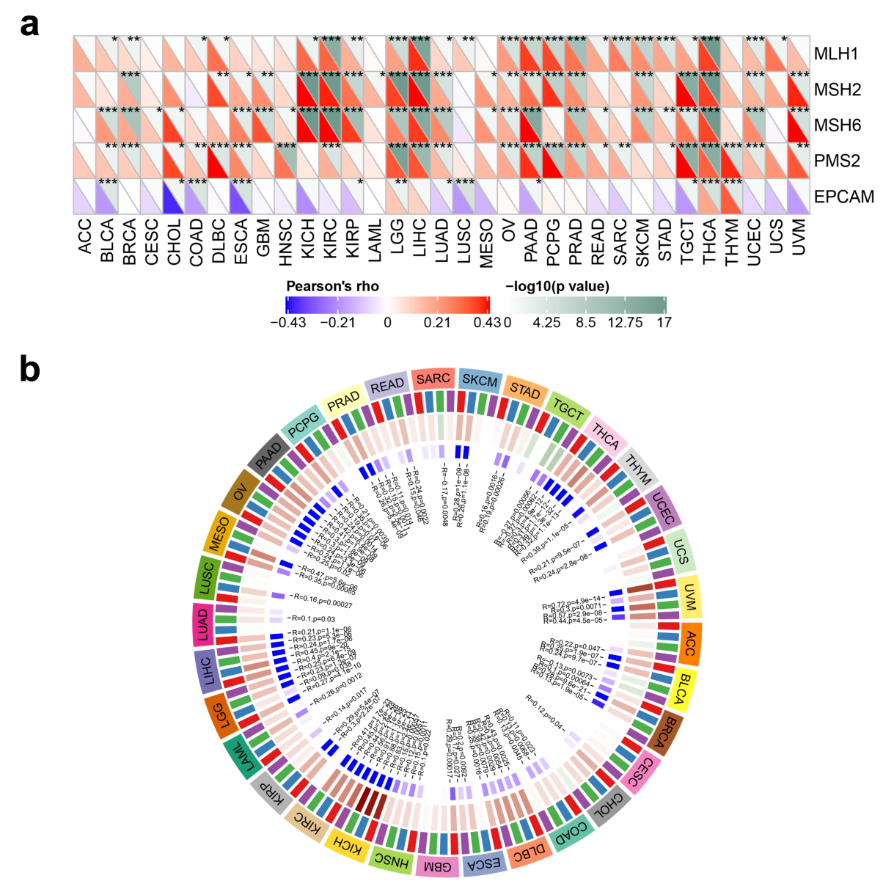


**Supplementary Figure 3.** Correlation of GPR176 with the MMR genes and DNA methylation. (a) Spearman correlation analysis of GPR176 expression with the expression of five MMR genes in pan-cancer; (b) Spearman correlation analysis of GPR176 expression with the expression of four methyltransferases in pan-cancer. Red represents DNMT1, blue represents DNMT2, green represents DNMT3A, and purple represents DNMT3B. (*P < 0.05, **P < 0.01, ***P < 0.001).
